# Guidelines for the Detection of a Common Source of Hepatitis B Virus Infections

**DOI:** 10.5812/kowsar.1735143X.773

**Published:** 2011-10-01

**Authors:** Mahmoud Reza Pourkarim, Marc Van Ranst

**Affiliations:** 1 Laboratory of Clinical Virology, Rega Institute for Medical Research, Catholic University of Leuven, Leuven, Belgium; 2 Blood Transfusion Research Center, High Institute for Research and Education in Transfusion Medicine, Tehran, IR Iran

**Keywords:** Transmission, Guidelines as Topic, Hepatitis B Virus

Hepatitis B virus (HBV) is an important threat to global public health. Despite the availability of safe and effective prophylactic vaccines, the existence of a large reservoir of chronic carrier patients impedes the eradication of this virus; therefore, HBV-related diseases still rank ninth on the global ranking of causes of mortality [1]. Moreover, HBV is considered the fifth most important infectious agent leading to death, with approximately 1 million HBV-related deaths occurring per year [2]. Previous studies showed that the infectivity rate of HBV is higher than those of other blood-borne viruses. The risk of infection after percutaneous exposure to the 3 major blood-borne viruses—HBV, hepatitis C virus (HCV), and human immunodeficiency virus (HIV)—varies greatly, and the risk of infection in non-immune individuals exposed to HBV may be over 30% if the source is hepatitis B e antigen (HBeAg) positive. The average infection rate for HCV and HIV is 1.8% and 0.3%, respectively [3]. The residual risk of pos  transfusion infection with HBV is higher than that with HIV and HCV [4]. Furthermore, the findings of recent studies indicate that HBV is more viable in needle syringes at room temperature than other blood-borne viruses [5]. The transmission pattern of HBV in different geographical regions depends on the local prevalence of chronic HBV carriers. For instance, in high prevalence (prevalence rate > 8%) regions such as South Asia, mother-to-child (vertical) transmission is the main route of transmission; in European countries with a low prevalence (prevalence rate < 2%), sexual transmission and unsafe injection practices are the main modes of HBV transmission. Nosocomial transmission and unsafe injection practices are responsible for more than 60% of HBV infections in Central Europe [6]. The main mode of HBV transmission in East Asia, a highly endemic region, is vertical. In West Asia and the Middle East, the route of transmission and HBV seroprevalence are variable depending upon the region. For instance, Iran  as low HBV endemicity (around 2%), and intravenous drug injections, tattooing, and phlebotomy are considered major potential risk factors and transmission routes of HBV infection in the country [7]. Further, socioeconomic statuses, life styles, occupations, and cultural attitudes in different ethnic groups greatly impact the route of HBV transmission in Iran [8]. During the last decade, several HBV outbreaks caused by nosocomial and iatrogenic transmissions have been reported in developed countries [9][10][11][12][13][14][15][16]. These findings indicate that several routes of transmission (i.e. multidose vials, dental equipment, dialysis units, finger-stick sampling, HBV-infected surgeons, and transplanted organs) exist despite efforts to educate health care workers about the risks of HBV transmission [17]. Nosocomial outbreaks occur more frequently in countries with moderate or high prevalence of HBV; however, these problems maybe missed or go unreported, because of weak monitoring and poor laboratory and surveillance facilities  at follow-up. Investigation of nosocomial outbreaks is an important issue in public health surveillance and should be conducted using accurate and sensitive strategies [18]. Identifying the common source of an HBV outbreak is a critical step in the investigation of nosocomial outbreaks, and accurate and extensive sampling is a prerequisite for such investigations. Good laboratory practices and reliable in silico analytical methods are also essential for successful investigations. Phylogenetic analysis of HBV nucleotide sequences can provide evidence for a common source in some nosocomial outbreaks [19][20]. In other reports on suspected nosocomial outbreaks, the investigators showed that the isolated strains were genetically unrelated and originated from different sources of infections (21, 22). Recently, a large nosocomial outbreak of HBV was reported in Belgium. In a retrospective cohort study, phylogenetic analysis of the fulllength genomes of 32 isolates led to the identification of a common HBV source. I  this case-control study, it was also investigated if the sequencing of partial HBV genomes (PreS1/S2 [± 520 nucleotides] and PreS1/S2/S [large S, ± 1200 nucleotides]) would have enough discriminatory power to accurately identify the common source. In both the phylogenetic trees constructed using partial genomes, a number of control sequences co-branched with the HBV isolated from the patient group. These results showed that partial sequences could not be used to appropriately reconstruct the transmission history and produced less accurate results than those produced by full-length genome analyses (20). In a previous study, we showed that the use of partial genome information for sub-genotyping might lead to misclassification of HBV subgenotypes [23]. In several cases, only partial genome data or a very small number of isolates were analyzed to find a common source of infection. In these cases, there is a high possibility that the datasets did not contain sufficient data to guarantee a sensitive analysis [24][25]. Although the cost of HBV whole genome sequencing may be a prohibitive factor in some settings, full-length HBV genome analysis is necessary for thorough investigations. In the molecular investigation of HBV outbreaks, the preferred approach is to compare HBV strains from the outbreak with HBV isolates from a control group. This control group should be chosen from HBV-infected subjects living in the same geographical region who had no previous contact with the presumed source of infection. These 2 groups should be phylogenetically analyzed together with separate full-length reference genomes retrieved from the GenBank database. A common source of infection is likely when the all the HBV strains of the outbreak cluster in a separate exclusive branch with a high bootstrap value and when the control and reference strains are distributed in different branches of the phylogenetic tree. In addition to phylogenetic analyses, other evolutionary tests should also be applied. It is useful to calculate the genetic distances between sequences in the HBV outbreak strain collection and in the control group. If the outbreak has a common source of infection, only a limited number of nucleotide variations will be observed among the sequences of the outbreak group and a much higher genetic divergence will be observed among the control samples. In addition, other evidences like a statistical model of distribution for infected cases over a period could support arguments for or against the existence of a common source. In conclusion, we would like to highlight some important steps to investigate an HBV outbreak, and either confirm or refute a common source of an HBV infection:

1. The study should be designed as a cohort study.

2. The control cases should be selected from HBV-infected patients living in the same geographical region who had no previous contact with the presumed source of infection.

3. Phylogenetic analysis should be conducted using a sufficiently large number of complete HBV genomes.

4. The genetic distances between  equences in the control group and the outbreak group should be calculated.

5. Additional analyses such as investigation of the distribution model of HBV-infected cases in the population over a period can help corroborate the existence of a common source of infection.
